# Application of multi-modality MRI-based radiomics in the pre-treatment prediction of RPS6K expression in hepatocellular carcinoma

**DOI:** 10.1186/s43556-023-00133-3

**Published:** 2023-07-24

**Authors:** Fan Yang, Yidong Wan, Xiaoyong Shen, Yichao Wu, Lei Xu, Jinwen Meng, Jianguo Wang, Zhikun Liu, Jun Chen, Di Lu, Xue Wen, Shusen Zheng, Tianye Niu, Xiao Xu

**Affiliations:** 1 Key Laboratory of Integrated Oncology and Intelligent Medicine of Zhejiang Province, Department of Hepatobiliary and Pancreatic Surgery, Affiliated Hangzhou First People’s Hospital, Zhejiang University School of Medicine, Hangzhou, 310006 China; 2 Institute of Translational Medicine, Zhejiang University School of Medicine, Hangzhou, 310020 Zhejiang China; 3 Department of Radiation Oncology, Sir Run Run Shaw Hospital, Zhejiang University School of Medicine, Hangzhou, 310016 Zhejiang China; 4 Department of Radiology, The First Affiliated Hospital, Zhejiang University School of Medicine, 79 Qinchun Road, Hangzhou, 310003 China; 5 Department of Pathology, The First Affiliated Hospital, Zhejiang University School of Medicine, 79 Qinchun Road, Hangzhou, 310003 China; 6 NHC Key Laboratory of Combined Multi-Organ Transplantation, Hangzhou, 310003 China; 7 Institute of Organ Transplantation, Zhejiang University, Hangzhou, 310003 China; 8 Department of Hepatobiliary and Pancreatic Surgery, Shulan Health Hangzhou Hospital, Hangzhou, 310004 Zhejiang China; 9 Institute of Biomedical Engineering, Shenzhen Bay Laboratory, Shenzhen, China; 10 Westlake Laboratory of Life Sciences and Biomedicine, Hangzhou, 310024 China

**Keywords:** RPS6K expression, Hepatocellular carcinoma, Magnetic resonance imaging, Radiomics, Machine learning

## Abstract

In this study, we aim to develop and validate a radiomics model for pretreatment prediction of RPS6K expression in hepatocellular carcinoma (HCC) patients, thus helping clinical decision-making of mTOR-inhibitor (mTORi) therapy. We retrospectively enrolled 147 HCC patients, who underwent curative hepatic resection at First Affiliated Hospital Zhejiang University School of Medicine. RPS6K expression was determined with immunohistochemistry staining. Patients were randomly split into training or validation cohorts on a 7:3 ratio. Radiomics features were extracted from T2-weighted and diffusion-weighted images. Machine learning algorithms including multiple logistic regression (MLR), supporting vector machine (SVM), random forest (RF), and artificial neural network (ANN) were applied to construct the predictive model. A nomogram was further built to visualize the possibility of RPS6K expression. The area under the receiver operating characteristic (AUC) was used to evaluate the performance of diagnostic models. 174 radiomics features were confirmed correlated with RPS6K expression. Amongst all built models, the ANN-based hybrid model exhibited best predictive ability with AUC of 0.887 and 0.826 in training and validation cohorts. ALB was identified as the key clinical index, and the nomogram displayed further improved ability with AUC of 0.917 and 0.845. In this study, we proved MRI-based radiomics model and nomogram can accurately predict RPS6K expression non-invasively, thus providing help for clinical decision making for mTORi therapy.

**Supplementary Information:**

The online version contains supplementary material available at 10.1186/s43556-023-00133-3.

## Key points

- RPS6K expression can reflect mTOR pathway on/off status, which is essential for mTOR inhibitor (mTORi) therapy. However, there is no non-invasive and accurate tool available for RPS6K expression in practice.
- Utilizing radiomics and machine learning algorithms, we explored and validated the feasibility of T2 phase- and DWI phase-derived high-dimension features for pretreatment prediction of RPS6K expression in HCC patients.

**Supplementary Information:**

The online version contains supplementary material available at 10.1186/s43556-023-00133-3.

## Introduction

Liver cancer, particularly hepatocellular carcinoma (HCC), is responsible for a significant amount of cancer-related mortality worldwide [1]. Despite advancements in surgical techniques and targeted therapies, the outlook for HCC patients remains bleak [2, 3]. However, recent research has highlighted the crucial role of molecular alterations and activation of specific oncogenic pathways in the development of liver cancer [4–7]. In this article, we discuss the significance of visualizing these key molecules and how it can aid in the precision management of liver cancer.

Early studies by Boyault S, et al. proposed a transcriptomic signature that classified HCC into six subgroups based on gene expression patterns. Notably, specific activation of the Akt pathway was detected in G1 and G2 HCCs, indicating the potential for Akt pathway-related therapy decisions [8]. The PI3K-Akt-mTOR signaling pathway is frequently activated in about 50% of HCCs [9–12]. Previous studies have demonstrated overexpression of the mTOR pathway in multinodular HCC and its correlation with increased post-liver transplantation (LT) HCC recurrence [13]. Villanueva et al. [14] found that chromosomal gains in RICTOR and positive p-RPS6 are associated with HCC recurrence. Studies have shown promising results with the early combination of everolimus and sorafenib in liver transplantation for HCC, confirming the potential of mTOR inhibitors (mTORi) for improving patient outcomes [15, 16]. However, the response to mTORi treatment varies widely in liver transplantation for HCC, emphasizing the need for a reliable classifier for mTOR activation in HCCs. The ribosomal protein S6 kinase (RPS6K) is the downstream effector of mTOR signaling and is considered a molecular surrogate of mTOR activation [17]. The current pretreatment confirmation of RPS6K expression relied on immunohistochemistry staining (IHC) on fine-needle aspiration (FNA) extracted specimens. As an invasive approach, FNA may result in severe complications. In addition, the very limited sample volume cannot reflect the heterogeneous background of the whole tumor, thus the FNA is limited in practice.

Radiomics, generally known as the high-throughput and quantitative feature mining from medical images and subsequent analysis [18, 19], is becoming widely utilized in cancer research, including tumor subtype diagnosis, gene/protein expression prediction, chemotherapy response evaluation, molecular phenotype classification and tumor microenvironment depiction [20–22].Based on the literature above, we thus hypothesized that: i) In HCCs, different expression levels of RPS6K were differently represented on pretreatment contrast-enhanced magnetic resonance imaging (CE-MRI) images in radiomics dimension; ii) both the expression level and expression difference of RPS6K in HCCs can be quantifiably predicted based on the pretreatment CE-MRI.

Herein, starting from a retrospective way and with multiple machine learning algorithms applied, we developed and validated contrast-enhanced MRI (CE-MRI)-based radiomics method for non-invasive and accurate prediction for RPS6K expression in HCC patients.

## Results

### Clinicopathological features of the study population

A total of 147 patients (male versus female: 126:21; mean age, 56.46 ± 10.85 years, ranging from 28 to 79 years) were enrolled. Among the included patients, 58 patients were determined as RPS6K-High group, while 89 were RPS6K-Low. As shown in Table 1, we found that tumor size, serum platelet, and serum albumin level were significantly related to RPS6K expression.

**Table 1:** Clinical characteristic of study population

*Characteristic*	*RPS6K High(n* = *58)*	*RPS6K Low* *(n* = *89)*	*P-value*
***Age***			0.784
≥ *50*	43	69	
< *50*	15	20	
***Gender***			0.918
*Male*	50	76	
*Female*	8	13	
***Differentiation***			0.612
*Good*	27	46	
*Poor*	31	42	
***Tumor size***			**0.020**
≥ *5 cm*	36	72	
< *5 cm*	22	17	
***Tumor number***			0.979
*Single*	41	64	
*Multiple*	17	25	
***Microvascular invasion***			0.292
*Yes*	24	46	
*No*	34	43	
***Macrovascular invasion***			0.996
*Yes*	20	32	
*No*	38	57	
***Lymph node metastasis***			0.067
*Yes*	0	3	
*No*	58	86	
***Cirrhosis***			0.098
*Yes*	46	58	
*No*	12	31	
***HBV infection***			0.623
*Yes*	38	63	
*No*	20	26	
***PLT***	150.5(99.25,188)	165(124,219)	**0.030**
***ALB***	39.85(35.325,42.475)	42.5(38.7,44.9)	** < 0.001**
***ALT***	29.5(20,42.25)	32(21,48)	0.0643
***AST***	33(24,53)	32(25,47)	0.0704
***GGT***	64.5(35.25,104.75)	83(47,163)	**0.042**
***FBG***	4.875(4.4375,5.785)	4.95(4.48,5.65)	0.470
***TB***	14(10,18)	14(11,16.5)	**0.047**
***DB***	5(3,8.675)	5(4,6)	**0.035**
***IB***	8(6,10.075)	9(7,10.2)	0.180
***Serum AFP***	45.1(6.25,737.175)	31.1(5.2,1905.9)	0.176
***Serum CEA***	2.5(1.75,3.75)	2.5(1.6,3.5)	0.509
***Serum CA19-9***	10(4.9,14.6)	8.7(4.7,16)	0.809

Continuous variables are expressed as mean ± standard deviation

Categorical variables given are the number of patients unless indicated otherwise

*HBV* Hepatitis B virus, *PLT* Platelet, *ALB* Albumin, *ALT* Alanine aminotransferase, *AST* Aspartate aminotransferase, *GGT* Gamma-glutamyl transferase, *FBG* Fasting blood glucose, *TB* Total bilirubin, *DB* Direct bilirubin, *IB* Indirect bilirubin, *AFP* α-fetoprotein, *CEA* Carcinoembryonic antigen, *CA19-9* Carbohydrate antigen 19-9. The tumor size were calculated by chi-square method. PLT, ALB, GGT, TB and DB were calculated by Mann-Whitney U test

### The extraction of radiomics feature correlated with RPS6K expression

A total of 174 radiomics features were found significant (Mann–Whitney U tests, two-sides *p* < 0.05), including 89 DWI and 85 T2 features in the training cohort. The optimal combination of complementary predictive features was chosen with the mRMR algorithm. The top ten important features were shown in Supplementary Fig. 2a. Interestingly, T2 features possessed a higher importance value than DWI features according to the mRMR importance ranking. This is probably due to the higher resolution of T2 Phase, rendering a better reflection of the tumor texture. We also observed that wavelet-based features played an important role in model construction. These features, extracted after decomposition by undecimated 3D wavelet transform, could further reflect the spatial heterogeneity at multiple scales within HCC.

### Development of radiomics model for RPS6K expression

**Fig. 1:**
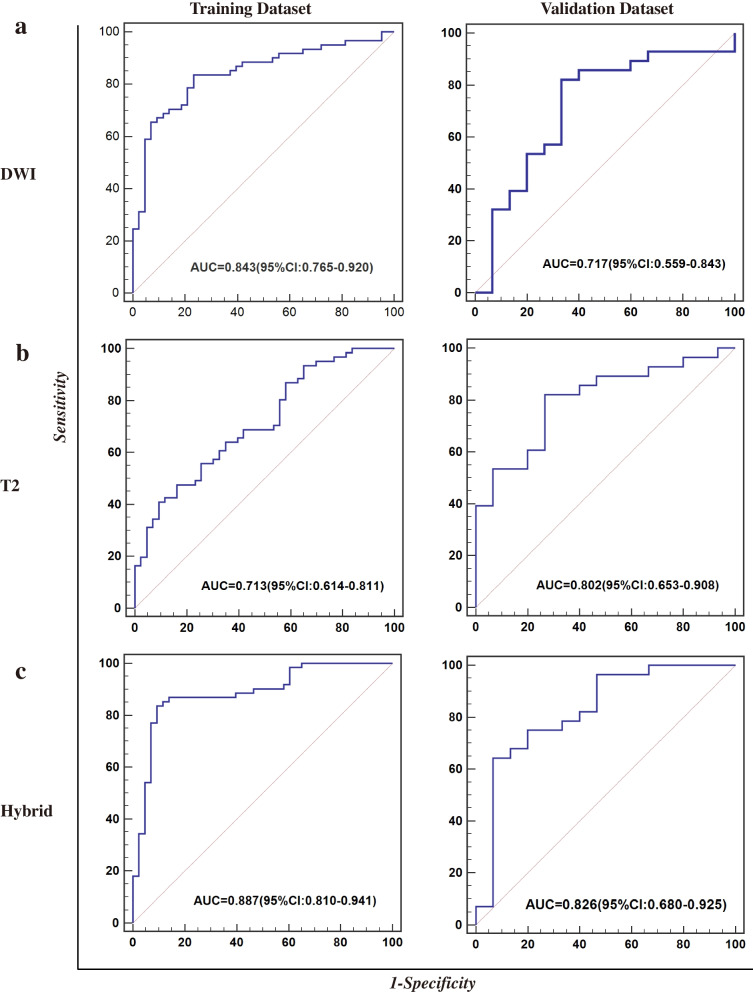
The performance of radiomics-based predictive models for RPS6K expression in the training set and the validation set: **a** DWI single-phase radiomics features; **b** T2 single-phase radiomics features; **c** Multi-modality Hybrid features

For the prediction model based on DWI features, the top-five mRMR-rank DWI features were selected through fivefold cross validation experiment, which were *DWI_RLN_LLH, DWI_Strength_HHL, DWI_GLN_LLL, DWI_contr* *,* *DWI_SZHGE.* And the ANN-based model exhibited the best performance with an AUC of 0.843 (95%CI: 0.765–0.920) for the training cohort and 0.717(95%CI: 0.559–0.843) for the validation cohort (Fig. 1a). For T2 phase features, the SVM-based model built with seven T2 features showed the best performance both in the training and validation cohort (training cohort: 0.713, 95%CI:0.614–0.811, validation:0.802, 95%CI: 0.653–0.908), as shown in Fig. 1b. The T2 features included *T2_SZLGE, T2ZSN_HHH, T2_autoc, T2_ZSN_HLL, T2_RLN_HLH, T2_cshad, T2_LGZE_HHH.* For the multi-modality features, the ANN-based hybrid model displayed the best performance among four algorithms. This model was developed with six radiomics features, which were *T2_SZLGE, DWI_RLN_LLH, T2ZSN_HHH, T2_autoc, T2_ZSN_HLL, DWI_Strength_HHL,* displayed with an AUC of 0.887 (95%CI: 0.810–0.941) for the training cohort and 0.826 (95%CI: 0.680–0.925) for the validation cohort (Fig. 1c). The hybrid model presented a better performance than other two single phase-based models (Supplementary Fig. 2b). The detailed description of radiomics features was listed in Supplementary Table 1. The performance of three other hybrid models developed by various methods in validation cohort were as follows: SVM-based model with an AUC of 0.790(95%CI:0.648–0.886), MLR-based model with an AUC of 0.790(95%CI:0.649–0.885), RF-based model with an AUC of 0.752(95%CI:0.596–0.861). The detailed performance of the T2 model and DWI model developed by other machine learning algorithms were shown in Supplementary Table 2.

### Development of the predictive nomogram for RPS6K expression

**Fig. 2:**
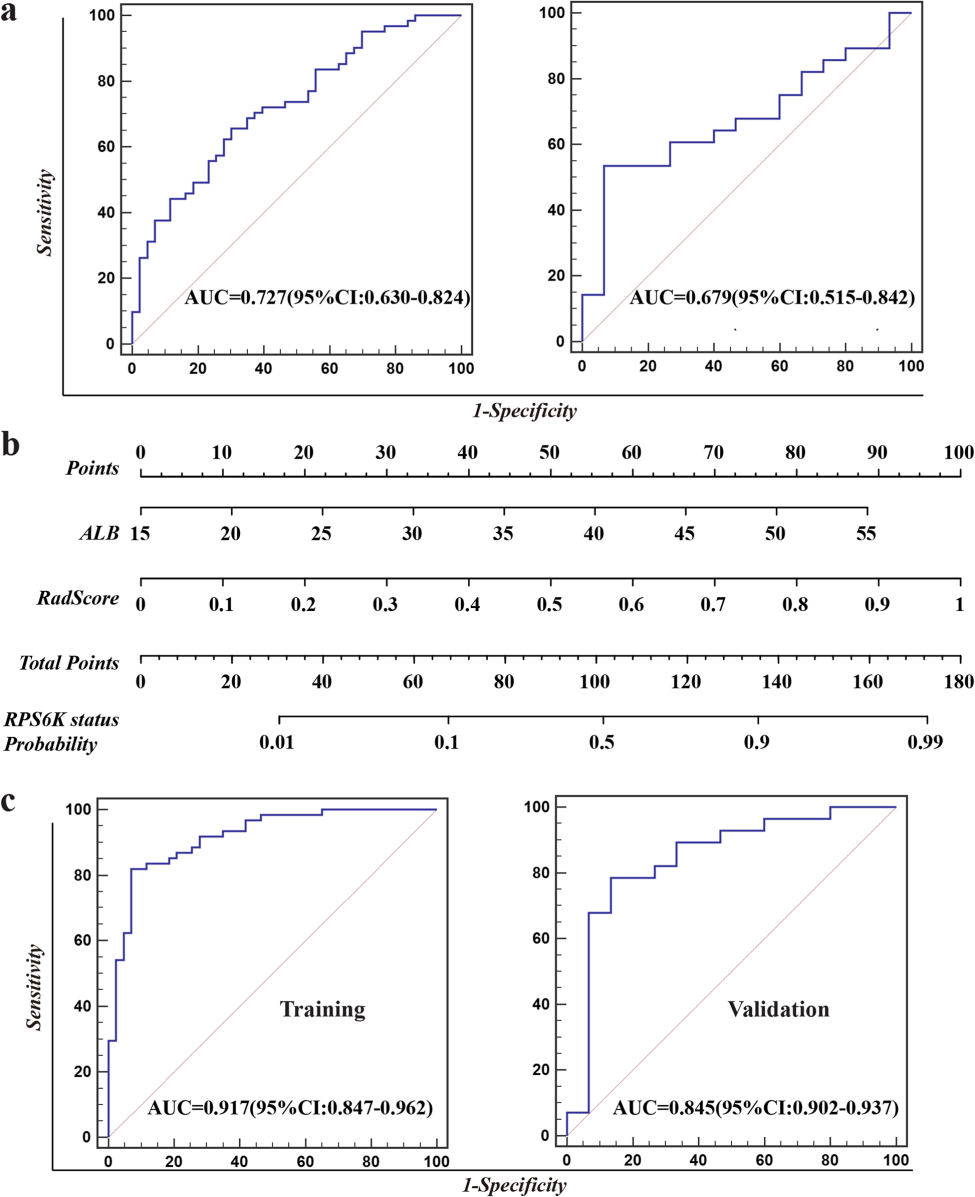
The clinical model and the nomogram for visualization of RPS6K expression in HCCs. **a** The performance of built clinical model is assessed with AUC; **b** The nomogram can help calculate and visualize RPS6K expression status; **c** The performance of built nomogram is assessed with AUC

**Fig. 3:**
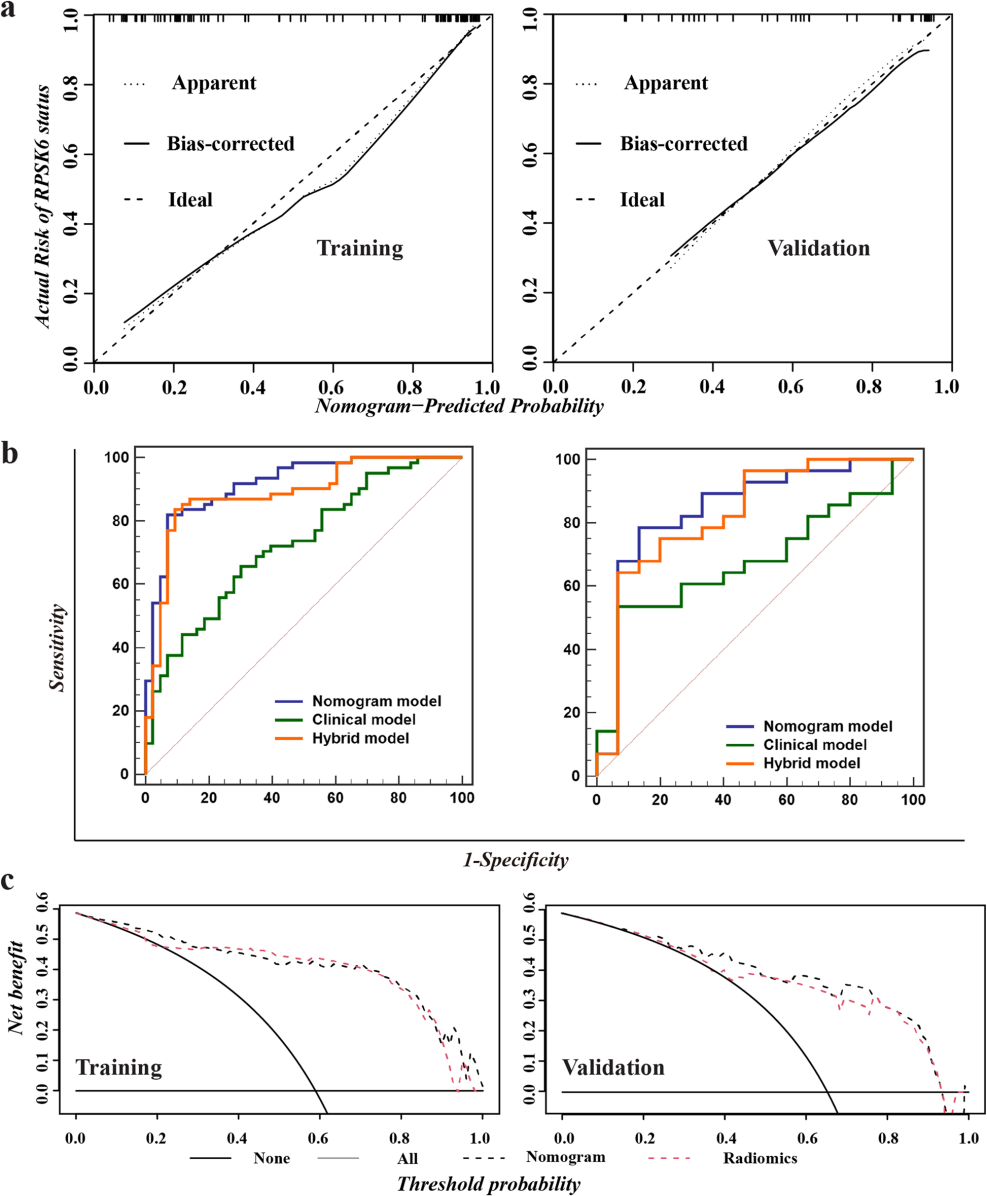
Performance comparison of built models and DCA analysis. **a** Calibration curve in the training and validation cohort. **b** The comparison of diagnostic ability of clinical model, radiomics model, and nomogram. **c** The clinical utility of the combined nomogram was evaluated by decision curve analysis (DCA). In the DCA analysis, the black dashed line indicated nomogram, and the red dashed line indicated hybrid radiomics models. In the comparison plot, the blue line indicated nomogram, the orange line indicated hybrid model, and the green line indicated clinical model

Six clinical indices were selected (Mann–Whitney U or Chi-suqare test, two-sided *p* < 0.05), including tumor size, PLT, ALB, GGT, TB, and DB. The clinical model was constructed with multiple logistic regression algorithm, exhibiting the lowest AIC value of 141.4, and a mediocre diagnostic ability (training cohort: 0.727, 95% CI: 0.630–0.824; validation cohort: 0.679, 95% CI: 0.515–0.842, Fig. 2a). Then we conducted the multivariable analysis founding that the model incorporating the ALB with RadScore displayed the lowest AIC (value = 73.8) score. Next, we explored the possibility to construct a nomogram with ALB and RadScore, and the nomogram demonstrated the best performance among all built models, with an AUC of 0.917 (95%CI: 0.847–0.962) in the training cohort and 0.845 (95%CI: 0.702–0.937) in the validation cohort, as shown in Fig. 2b-c. The calibration curves for the combination nomogram were shown in Fig. 3a. In both training and validation cohorts, non-significant statistic deviations (training cohort: *p* = 0.4983; validation cohort:* p* = 0.4473) were determined with the H–L test, indicating the goodness-of-fit of the models. The AUC comparison showed that the nomogram outperformed the hybrid radiomics model and the clinical model (Fig. 3b). However, Delong-test showed that non-significance between the nomogram, clinical model, and radiomics model in both training and validation cohorts, indicating though the improvement was achieved, it was not significant. The clinical utility of the combined nomogram was evaluated by decision curve. As the figure is shown in Fig. 3c, the combined nomogram (black lines) showed a more promising clinical utility than the hybrid radiomics model (red line).

## Discussion

HCC is a highly heterogeneous entity with frequent molecular alteration. To the credit of the development of next-generation sequencing technology, the crucial role of molecular heterogeneity in hepatocarcinogenesis is becoming much more noted. Previous studies demonstrated that the mTOR pathway was highly activated in HCC tissues, cirrhotic liver tissues from HCC patients, high-grade HCC, and poorer prognostic subtype [23–25]. Under this background, researchers attempted to classify HCCs into different subtypes based on molecular mutation to guide clinical decision making, like G1-G6 classification and S1-S3 classification. It has been noted that inhibition of the mTOR pathway could prevent HCC progression [26] and protect the liver from irradiation-induced damage for those with recurrent HCC [27]. Evidence from clinical trials indicated that Sirolimus use could improve recurrence-free survival (RFS) and overall survival (OS) of LTx for HCC [28, 29]. As mutation frequency of the mTOR pathway in HCC patients is about 50%, the mTORi therapy is not appropriate for all, and it’s necessary to identify the mutation status of the mTOR pathway in HCC patients before mTORi application. However, current confirmation of mTOR pathway in HCCs relies on IHC staining of FNA-extracted specimens or resected specimens on specific key molecules invloed in mTOR pathway, like pMTOR and RPS6K. As an invasive method, FNA can result in hemorrhage and abdominal tumor planting. For patients with large HCC, it can lead to tumor rupture and thus highly risky and inappropriate. In addition, considering the heterogeneity background, the limited specimen volume may not be enough, and the information can be misleading. In general, there is no available method to get whole-scale heterogeneity of HCC. Therefore, it is important to develop a practical method that is non-invasive, accurate, and capable of reflecting the whole-scale heterogeneity of HCC.

The emergence of Radiomics provides the possibility to grasp the full-view heterogeneity of HCC in a non-invasive and accurate way [30, 31]. It’s been proved that with radiomics features extracted, deciphering tumor characteristics from the macro-to-micro level is no longer a fairy tale [32]. After a comprehensive literature retrieval, we found there was no available radiomics-based method to determine mTOR signaling activation status or mTOR pathway key molecule expression prediction. Integrating artificial intelligence, i.e. machine learning and deep learning, it is promising to fill the exited gap between bench to clinical practice. Thus, we carried out this pilot study from a retrospective angle, integrating clinical parameters, CE-MRI radiomics features, and machine learning algorithms to develop the pre-treatment classifier, guiding mTORi selection in HCCs management regarding different RPS6K expression level RPS6K expression and low expression from a radiomics perspective.

Of the six significant clinical indices, the combination of ALB, TB, and GGT was identified as the best indices. The clinical model achieved an AUC of 0.727 and 0.679 in the training and validation cohort, respectively, which was only mediocre in our opinion. We next assessed the performance of radiomics-based methods. 174 features were significantly different between opposite RPS6K statuses. The mRMR-based importance ranking was applied to identify the most critical features of the single-phase (DWI/T2) and hybrid phases. To avoid the bias deriving from the single algorithm, we utilized MLR, SVM, RF, and ANN algorithms in model construction (shown in Supplementary Table 2). We compared the diagnostic ability between the single phage (DWI/T2) radiomics model and the hybrid (DWI and T2) model. Both single-phase (DWI/T2) radiomics models outperformed the clinical model, and the T2 feature-based model showed a slightly better performance than DWI feature-based model. The hybrid model, integrating both T2 and DWI features, displayed better diagnostic performance than single-phase features-derived models, achieving an AUC of 0.887 and 0.826 in the training and validation cohorts. We then questioned whether adding a clinical index could further improve the diagnostic ability. As expected, with the addition of ALB, the nomogram yielded much-improved performance, with an AUC of 0.917 and 0.845 in training and validation cohorts, respectively. All these results answered that the MRI-radiomics difference did exist between HCC patients of opposite RPS6K status and strongly demonstrated that MRI-based radiomics models could help clinical evaluation of RPS6K expression of HCC patients in a non-invasive and accurate fashion, further providing pretreatment evidence for mTORi therapy.

Some limitations of this study should be noted. First, as this study was retrospective, potential selection bias might exist, thus hampering the results' reproducibility and comparability. Second, the study consisted of a relatively small research scale from a single center, which limited the universal value of the study results. To further improve the reliability of the results, the spatial universality, the clinical translational value, and a larger sample scale from independent medical centers were necessarily required in further studies. During the nomogram model construction stage, we could further investigate the fusion model based on both radiomics features and clinical factors to enhance the accuracy of model and we could try to make our propose more interpretable.

In conclusion, with multiple machine learning algorithms applied, we successfully developed and validated MRI-radiomics models, both single-phase and multi-modality, and the nomogram for RPS6K expression in HCC patients in a non-invasive and accurate manner. Our research can provide informative clues for mTORi therapy management.

## Materials and methods

### Study population

**Fig. 4:**
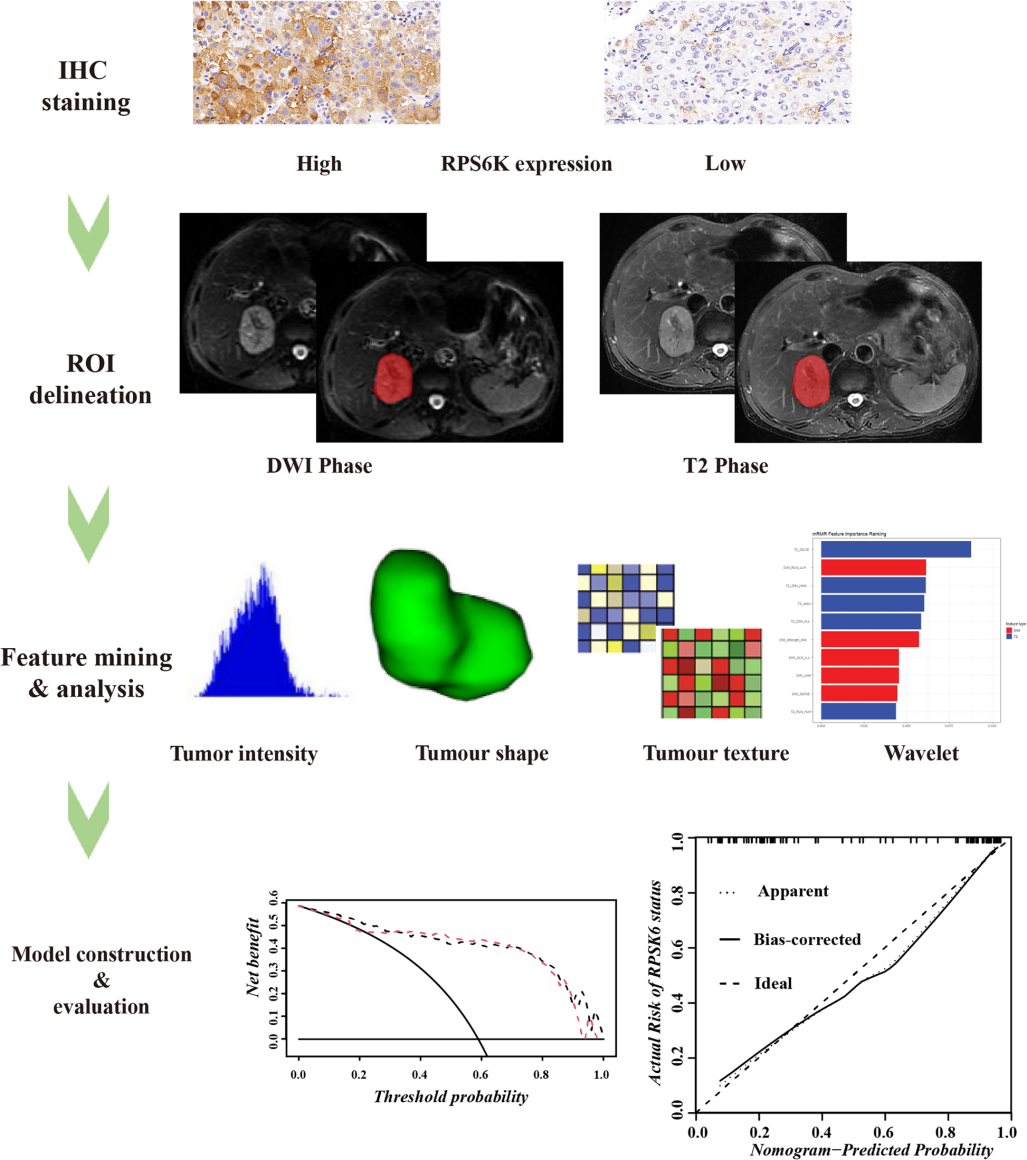
The overall workflow of the proposed study. The process includes four main steps: (1) Determination of RPS6K expression in HCCs based on IHC staining; (2) Delineation of ROI in studied MRI phases; (3) Extraction of radiomics features and further analysis; (4) Predictive models construction and evaluation

The study retrospectively enrolled and evaluated patients diagnosed with liver mass and treated from January 2018 to May 2019 in First Affiliated Hospital Zhejiang University School of Medicine (FAH-ZJU). The overall workflow of the proposed study was shown in Fig. 4. Based on the IHC score of RPS6K, enrolled patients were categorized into RPS6K high and low groups. For model construction and validation, patients were randomly divided into the training cohort (*n* = 104) and validation cohort (*n* = 43) on a ratio of 7:3.

The ethical approval of this study was obtained from the Human Research Ethics Committee (HREC) of FAH-ZJU (No.2018768). Informed consent from patients was waived for the retrospective usage of their medical images, clinical information and biospecimen. All the identities information were recoded to protect patients’ privacy. The inclusion criteria were as follows: (1) complete pre-surgery gadoxetic acid enhanced MRI (within two weeks); (2) paraffin-embedded surgical tumor biospecimen; (3) complete clinical information. The exclusion criteria were as follows: (1) distal metastasis or concurrent malignancies; (2) unqualified MR images, i.e., broken, missing, or strongly affected by artificial crafts; (3) small tumor, i.e., tumor size smaller than 2 cm on the maximum diameter; (4) incomplete clinical information. The patient recruitment process and inclusion/exclusion criteria were shown in Supplementary Fig. 1.

Baseline clinical and histopathological characteristics were as follows: age, gender, HBV infection status, serum liver-kidney function, serum tumor markers (AFP, CEA, CA19-9), liver cirrhosis, tumor maximum diameter, tumor number, tumor differentiation grade, microvascular invasion, lymph node metastasis, and distal metastasis. All biochemical blood tests were performed before surgery.

### Immunohistochemistry staining

A tissue microarray containing 147 HCC samples. In brief, following histopathological examination, a single core with a 2.0-mm diameter was extracted from each sample and placed on a tissue array (Xinchao Company, Hangzhou). The IHC staining for RPS6K was conducted according to the manufacturer’s protocols (*Cell Signaling Technology, Phospho-S6 Ribosomal Protein, Ser235/236, D57.2.2E*). Protein expression levels were evaluated based on the staining intensity (scored as 0 points for pale, 1 point for mild, 2 points for moderate, and 3 points for intense) and the percentage of positive cells relative to the total cells in each field (scored as 0 points for < 1%, 1 point for < 25%, 2 points for 25–50%, 3 points for 50–69%, and 4 points for ≥ 70%). Protein expression was expressed as the multiplied score, which was calculated as $$multiplied\ score=intensity\ of\ staining*percentage*100\%$$. The final score for each patient was determined based on the consensus of two pathologists, who were kept blinded to the clinicopathological information. The median value of the multiplied score was chosen as the cut-off value for RPS6K status grouping.

### Image acquisition

The pretreatment MR images were obtained from the Picture Archiving and Communication Systems (PACS) of FAH-ZJU. All MR examination was carried out with GE SIGNA Architect 3.0 T. Patients fasted for 4–6 h before the scan, which was performed after the injection of gadoxetic acid (0.025 mmol/kg, *Consun GAPENSUANPU’AN ZHUSHEYE, 15 mL:7.04 g, Guangzhou Consun Pharmaceutical Co., Guangzhou, China*) at a rate of 2 mL/s via an injector. Then 20 mL saline was injected at the same rate. The signal of HCC is lower than the normal liver, while in T2 phase, the signal of HCC is higher. In DWI phase, the signal of HCC is higher even when the lesion is too small to be diagnosed in other phases. From our previous experience, delineation of the HCC lesion is much easier and timesaving in T2 and DWI phases. Thus, T2 and DWI phase are selected for further radiomics research. The detailed information of DWI and T2 Phase were described in the Supplementary Materials.

### Regions of interest segmentation

**Fig. 5:**
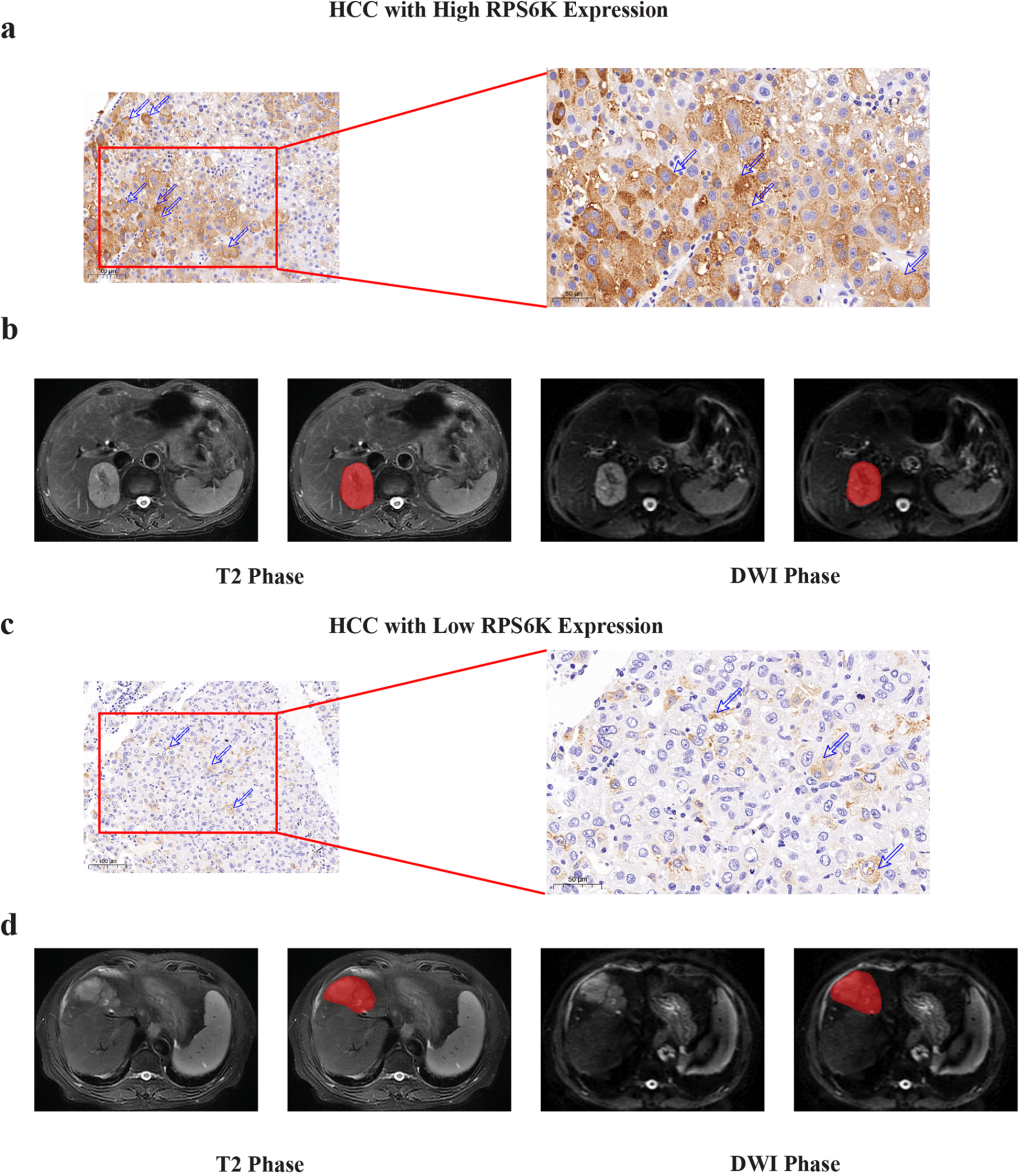
Typical IHC staining and delineation of the ROI on MRI of HCCs with different RPS6K expression. IHC staining based RPS6K expression (**a**, HCC with high RPS6K expression; **d**, HCC with low RPS6K expression). Undelineated and delineated image of contrast-enhanced T2-weighted imaging (**b**, high RPS6K expression; **e**, low RPS6K expression). Undelineated and delineated image of contrast-enhanced diffusion-weighted imaging (**c**, high RPS6K expression; **f**, low RPS6K expression)

The open-source software ITK-SNAP (http://www.itksnap.orge) was utilized to perform the region of interest (ROI) contour [33], as shown in Fig. 5. ROIs were manually contoured by two senior radiologists specializing in abdomen diagnosis on studied phases. A chief radiologist further performed a final check on all segmentation.

### Radiomics feature extraction

Radiomics features were extracted from delineated tumor ROI on both phases. This process was conducted using an open source radiomics package in MATLAB 2016a software (MathWorks, Natick, MA, USA). The toolkit could be found and installed form the Add-On Explorer of MATLAB or be achieved by an open source (https://github.com/mvallieres/radiomics). All images were resampled to the same voxel size of $1\times1\times1{mm}^3$ using the bicubic interpolation method. The intensity was discretized to the same range from 1 to 64 intensity values to decrease the difference among all image scans. 547 radiomics features were extracted from each ROI from the 3D quantitative image feature pool, including 7 shape features, 22 histogram features, 22 Gy-level co-occurrence matrix (GLCM) features, 13 Gy-level run-length matrix (GLRLM) features, 13 Gy-level size zone matrix (GLSZM) features, 5 neighborhood gray-tone difference matrix features, and 480 wavelet-based features. Two modalities of MR images were involved in this study, so a total of 1094 radiomics features were collected from each patient. To account for differences in scale between various features, all radiomics features were normalized to a Z-score by subtracting the mean and dividing by the standard deviation. The mean and standard deviation of each feature in the training cohort were used to normalize the corresponding features in the validation cohort.

### Radiomics features selection and construction

Due to the large number of extracted features, Mann–Whitney U tests and Max-Relevance and Min-Redundancy (mRMR) algorithm were used in the training cohort to perform the features dimension reduction and feature selection. First, features with a two-sided *p-value* less than 0.05 were kept. Second, the most relevant features to the status of RPS6K expression and features that had minimum redundancy were retained, the specific number of radiomics features was determined by conducting a fivefold cross-validation experiment in the training cohort, and the number of features was set from three to ten. In the internal validation cohort, the optimal feature subset was identified based on the highest AUC value. Multiple logistic regression (MLR), support vector machine (SVM) with a gaussian kernel, random forest (RF), and artificial neural network (ANN) algorithms were utilized to construct the model and further compared to determine which possessed the best ability in the validation cohort. The performance of different models (single-phase (DWI/T2) radiomics model and multi-modality hybrid radiomics model) were further evaluated by AUC. 95% confident confidence interval (CI) was also recorded. The hybrid radiomics model calculated the radiomics score (RadScore) corresponding to the predicted value for each patient.

### Construction of clinical model and combined nomogram model

We first built a clinical index-based model for the expression of RPS6K. To further evaluate the potential additive value of clinical factors, the selected clinical index and RadScore were used to construct a predictive nomogram. We employed a two-step feature selection strategy to obtain the most relevant clinical factors, i.e., univariate analysis and multivariable analysis approach. First, clinical factors used Mann–Whitney or chi-square test with a two-sided *p-value* less than 0.05 were selected. The backward search method, using Akaike Information Criterion (AIC) score, was employed to determine the optimal combination of variables for model development. This approach carefully evaluated the quality of the developed models, taking into consideration both the binomial deviance and the number of variables in the selection process [34]. Then, the combination with the lowest AIC score was chosen as the optimal combination for nomogram construction. The Delong-test method was used to evaluate the significance among different models [35]. Hosmer–Lemeshow (H–L) test was used to assess the goodness-of-fit of the nomogram. The calibration curve was applied to evaluate the performance of the nomogram. The degree of overlap between the calibration curve and the diagonal in the graph represented the predictive accuracy of the proposed nomogram. Decision curve (DCA) analysis was employed to assess the additive value of the clinical index. The threshold of predicted probability using the nomogram was plotted on the x-axis of the decision curve, while the y-axis showed the clinical decision net benefit for patients based on the classification result at that threshold. The decision curves for the treat-all scheme and the treat-none scheme were used as reference points in the DCA.

### Statistical analysis

For continuous variables, Mann–Whitney U tests were employed, while chi-square tests were used for qualitative variables. Radiomics features were all considered continuous variables, and twelve clinical factors (PLT, ALB, ALT, AST, GGT, FBG, TB, IB, serum AFP, serum CEA, and serum CA199) were also treated as continuous variables. Age, gender, differentiation, tumor size, tumor number, microvascular invasion, and lymph node metastasis were considered discrete variables. Mann–Whitney U tests were used for continuous variables, while chi-square tests were used for discrete variables. All statistical tests were two-sided, and a P-value less than 0.05 was considered statistically significant. Statistical analyses were performed using IBM SPSS Statistics 20 and R software (version 3.4.1; http://www.Rproject.org). Nomograms and calibration tests were created using the "rms" package. The calibration process used a resampling algorithm with 1000 random experiments and 50 samples in each resampling experiment to evaluate the proposed model. The "generalhoslem" package was used to assess the goodness of fit of logistic models using the Hosmer–Lemeshow test. In this study, the risk was split into ten quantiles, and a false ordinal logistic regression model was set for calibration. The AUC analysis was conducted using the "pROC" package, and DCA was performed using the "dca.R" function. Hyperparameters for model construction were determined through a fivefold cross-validation test conducted using the training cohort.

## Supplementary Information

**Additional file 1:Supplementary Table 1. Supplementary Table 2.** Details of MLR, SVM, RF, and ANN Algorithms Derived Models.

**Additional file 2:Supplementary Figure 1.** The overall enrollment of the whole study. 147 of 200 patients were eligible for the final analysis after assessment.

**Additional file 3:Supplementary Figure 2. **Radiomics feature selection strategy and comparison of radiomics models.

## Data Availability

The raw data of this manuscript is available by the corresponding authors to qualified researchers upon reasonable request.

## Abbreviations

AIC: Akaike information criterion
ALB: Albumin
ALT: Alanine aminotransferase
AST: Aspartate aminotransferase
ANN: Artificial neural network
AUC: Area under the receiver operating characteristic
AFP: Alpha-fetoprotein
DB: Direct bilirubin
DCA: Decision curve analysis
DWI: Diffusion weighted imaging
FBG: Fasting blood glucose
FNA: Fine needle aspiration
GGT: Gama-glutamyltranferase
GLCM: Gray-level co-occurrence matrix
GLRLM: Gray-level run-length matrix
GLSZM: Gray-level size zone matrix
LT: Liver transplantation
MLR: Multiple logistic regression
HCC: Hepatocellular carcinoma
H–L test: Hosmer–Lemeshow test
HBV: Hepatitis B virus
IB: Indirect bilirubin
IHC: Immunohistochemistry
mRMR: Maximum relevance minimum redundancy
mTORi: MTOR inhibitor
PACS: Picture archiving and communication system
RadScore: Radiomics score
RF: Random forest
RPS6K: Ribosomal protein S6 kinase
ROI: Region of interest
SVM: Supporting vector machine
TB: Total bilirubin
