# Supplementary material for: Application of multi-modality MRI-based radiomics in the pre-treatment prediction of RPS6K expression in hepatocellular carcinoma
Source: Mol Biomed. 2023 Jul 24;4:22. doi: 10.1186/s43556-023-00133-3 (PMC10363521; doi:10.1186/s43556-023-00133-3)
Supplement: Supplementary file 1 — Additional file 1:Supplementary Table 1. Supplementary Table 2. Details of MLR, SVM, RF, and ANN Algorithms Derived Models. [file 43556_2023_133_MOESM1_ESM.docx]

Supplementary Materials

Supplementary Tables

**Supplementary Table 1**

| **Feature Name** | **Formula** | **Explanation** |
| --- | --- | --- |
| *T2_SZLGE* | $\frac{\sum_{j=1}^{N_{g}} \sum_{j=1}^{N_{s}} \frac{P(i,j)}{i^{2}j^{2}}}{N_{z}}$ | SZLGE of T2 measures the proportion in the image of the joint distribution of smaller size zones with lower gray-level values. |
| *DWI_RLN_LLH* | $\frac{\sum_{j=1}^{N_{r}} {(\sum_{i=1}^{N_{g}} P(i,j\vert\theta))}^{2}}{N_{r}(\theta)}$ | RLN_LLH of DWI measures the similarity of run lengths throughout the image, with a lower value indicating more homogeneity among run lengths in the image by performing low-pass wavelet filter on x- and y- axis and high-pass wavelet filter on z-axis. |
| *T2_ZSN_HHH* | $\frac{\sum_{j=1}^{N_{s}} {(\sum_{i=1}^{N_{g}} P(i,j))}^{2}}{N_{z}}$ | ZSN_HHH of T2 measures the variability of size zone volumes in the image, with a lower value indicating more homogeneity in size zone volumes by performing high-pass wavelet filter on all 3 axes. |
| *T2_autoc* | $\sum_{i=1}^{N_{g}} \sum_{j=1}^{N_{g}} p(i,j)ij$ | Autocorrelation of T2 measures the magnitude of the fineness and coarseness of texture. |
| *T2_ZSN_HLL* | $\frac{\sum_{j=1}^{N_{s}} {(\sum_{i=1}^{N_{g}} P(i,j))}^{2}}{N_{z}}$ | ZSN_HLL of T2 measures the variability of size zone volumes in the image, with a lower value indicating more homogeneity in size zone volumes by performing low-pass wavelet filter on y- and z- axis and high-pass wavelet filter on x-axis. |
| *DWI_Strength_HHL* | $\frac{\sum_{i=1}^{N_{g}} \sum_{j=1}^{N_{g}} (p_{i}+p_{j}){(i-j)}^{2}}{\sum_{i=1}^{N_{g}} S_{i}}$ | Strength_HHL of DWI measures the primitives in an image. Its value is high when the primitives are easily defined and visible, i.e., an image with slow change in intensity but more large coarse differences in gray level intensities by performing high-pass wavelet filter on x- and y- axis and low-pass wavelet filter on z-axis. |
| *DWI_GLN_LLL* | $\frac{\sum_{i=1}^{N_{g}} {(\sum_{j=1}^{N_{d}} P(i,j))}^{2}}{N_{z}}$ | GLN_LLL of DWI measures the similarity of gray-level intensity values in the image, where a lower GLN value correlates with a greater similarity in intensity values by performing low-pass wavelet filter on all 3 axes. |
| *DWI_contr* | $(\frac{1}{N_{g.p}(N_{g.p}-1)}\sum_{i-1}^{N_{g}} \sum_{j=1}^{N_{g}} P_{i}P_{j}\left( i-j \right)^{2}(\frac{1}{N_{v,p}}\sum_{i=1}^{N_{g}} s_{i})$ | Contrast of DWI measures the spatial intensity change, but is also dependent on the overall gray level dynamic range. Contrast is high when both the dynamic range and the spatial change rate are high, i.e., an image with a large range of gray levels, with large changes between voxels and their neighborhood. |
| *DWI_SZHGE* | $\frac{\sum_{j=1}^{N_{g}} \sum_{j=1}^{N_{s}} \frac{P(i,j)}{i^{2}j^{2}}}{N_{z}}$ | *SZHGE* of DWI measures the proportion in the image of the joint distribution of smaller size zones with lower gray-level values. |
| *DWI_GLV_LLH* | $\sum_{i=1}^{N_{g}} \sum_{j=1}^{N_{r}} P\left( i,j \vert\theta\right){(i-\mu)}^{2}$  $\mu=\sum_{i=1}^{N_{g}} \sum_{j=1}^{N_{r}} P\left( i,j \vert\theta\right)i$ | GLV_LLH of DWI measures the variance in gray level intensity for the runs by performing low-pass wavelet filter on x- and y- axis and high-pass wavelet filter on z-axis. |
| *T2_RLN_HLH* | $\frac{\sum_{j=1}^{N_{r}} {((\sum_{i=1}^{N_{g}} P\left( i,j \vert\theta\right)))}^{2}}{N_{r}(\theta)}$ | RLN_HLH of T2 measures the similarity of run lengths throughout the image, with a lower value indicating more homogeneity among run lengths in the image by performing high-pass wavelet filter on x- and z- axis and low-pass wavelet filter on y-axis. |
| *T2_cshad* | $\sum_{i=1}^{N_{g}} \sum_{j=1}^{N_{g}} \left[ i+j-\mu_{x}(i)-\mu_{y}(j) \right]^{3}P(i,j)$ | Cshad of T2 measures the skewness and uniformity of the GLCM. |
| **Notation:**  $X$ denote the three-dimensional image matrix with $N$ voxels and $P$ the first order histogram divided by $N_{l}$ discrete intensity levels.  A Gray Level Co-occurrence Matrix (GLCM) of size $N_{g}\times N_{g}$describes the second-order joint probability function of an image region constrained by the mask and is defined as $P(i,j\vert\delta,\theta)$.  $\bar{X}$ is the mean of $X$  $N_{p}$ the number of voxels in the image.  $N_{r}$ the number of different size matrix  $N_{g}$be the number of discrete intensity levels in the image.  $I(i)$ is the gray level of the i-th voxel in the tumor region.  $H(i)$ is the number of voxels with gray-level i in the histogram of image.  $M(n,s)$ is the (n, s) th value of Gray-Level Size-Zone Matrix.  $N$ is the number of gray-level bins.  $S$ is the number of zone-size bins.  $P(i,j)$ be the co-occurrence matrix for an arbitrary $\delta$ and $\alpha$  $\mu$ be the mean of $P(i,j)$  $p_{x}\left( i \right)=\sum_{j=1}^{N_{g}} P(i,j)$ be the marginal row probabilities  $p_{y}\left( i \right)=\sum_{i=1}^{N_{g}} P(i,j)$ be the marginal column probabilities  $\mu_{x}$ be the mean of $p_{x}$  $\mu_{y}$ be the mean of $p_{y}$  $\sigma_{x}$ be the standard deviation of $p_{x}$  $\sigma_{y}$ be the standard deviation of $p_{y}$ | | |

**Supplementary Table 2 Details of MLR, SVM, RF, and ANN Algorithms Derived Models**

|  | Training cohort | Validation cohort |
| --- | --- | --- |
| Hybrid model | | |
| MLR | 0.763(95%CI:0.671-0.856) | 0.790(95%CI:0.649-0.885) |
| SVM | 0.759(95%CI:0.665-0.853) | 0.790(95%CI:0.648-0.886) |
| RF | 0.844(95%CI:0.767-0.921) | 0.752(95%CI:0.596-0.861) |
| **ANN** | **0.887 (95CI%: 0.810-0.941)** | **0.826 (95%CI: 0.680-0.925)** |
| DWI model | | |
| MLR | 0.703(95%CI:0.602-0.803) | 0.710(95%CI:0.559-0.827) |
| SVM | 0.691(95%CI:0.588-0.794) | 0.686(95%CI:0.530-0.808) |
| RF | 0.811(95%CI:0.729-0.894) | 0.700(95%CI:0.541-0.826) |
| **ANN** | **0.843(95%CI:0.765-0.920)** | **0.717(95%CI:0.559-0.843)** |
| T2 model | | |
| MLR | 0.725(95%CI:0.627-0.823) | 0.786(95%CI:0.633-0.894) |
| **SVM** | **0.713(95%CI:0.614-0.811)** | **0.802(95%CI:0.653-0.908)** |
| RF | 0.823(95%CI:0.744-0.903) | 0.736(95%CI:0.565-0.862) |
| ANN | 0.839(95%CI:0.762-0.916) | 0.755(95%CI:0.585-0.880) |

Scanner: GE Architect3.0

**DWI parameter:**

slice thickness：6.0mm

spacing：0.5mm

Acq Voxel Size：2.4×2.4×6.0

b-values：2 50 1000

Diffusion Direction：3in1

TE：minimum

Frequency：160

Phase：128

**T2 parameter:**

slice thickness：6.0mm

spacing：0.5mm

Acq Voxel Size：0.9×0.9×6.0

TE：85

Frequency：400

Bandwidth：100

Auto Refocus Flip Angle：111

Echo Train Length：32
