# Supplementary figures and images for: Application of multi-modality MRI-based radiomics in the pre-treatment prediction of RPS6K expression in hepatocellular carcinoma
Source: Mol Biomed. 2023 Jul 24;4:22. doi: 10.1186/s43556-023-00133-3 (PMC10363521; doi:10.1186/s43556-023-00133-3)

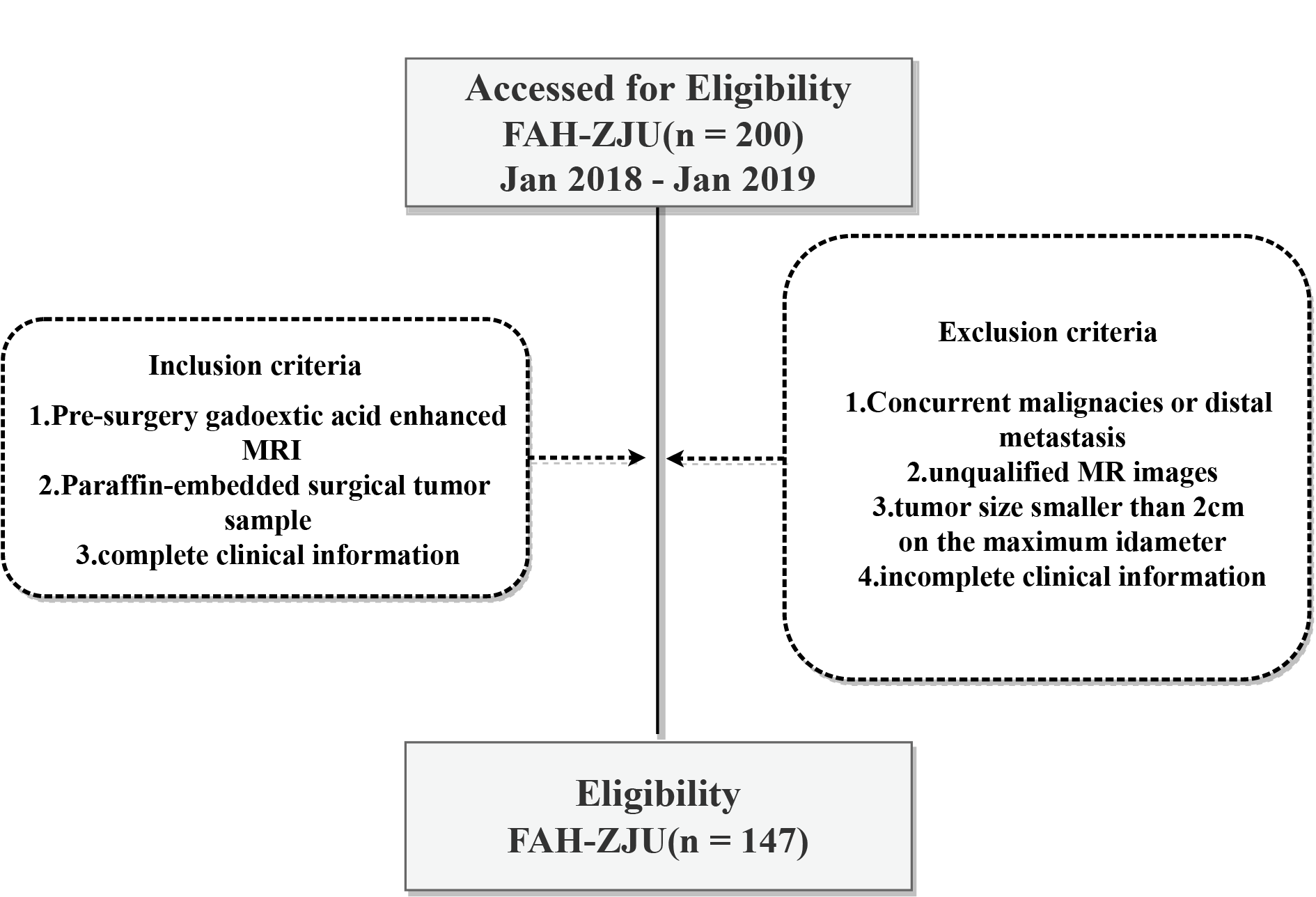

Supplement: Supplementary file 2 — Additional file 2:Supplementary Figure 1. The overall enrollment of the whole study. 147 of 200 patients were eligible for the final analysis after assessment. [file 43556_2023_133_MOESM2_ESM.tif]

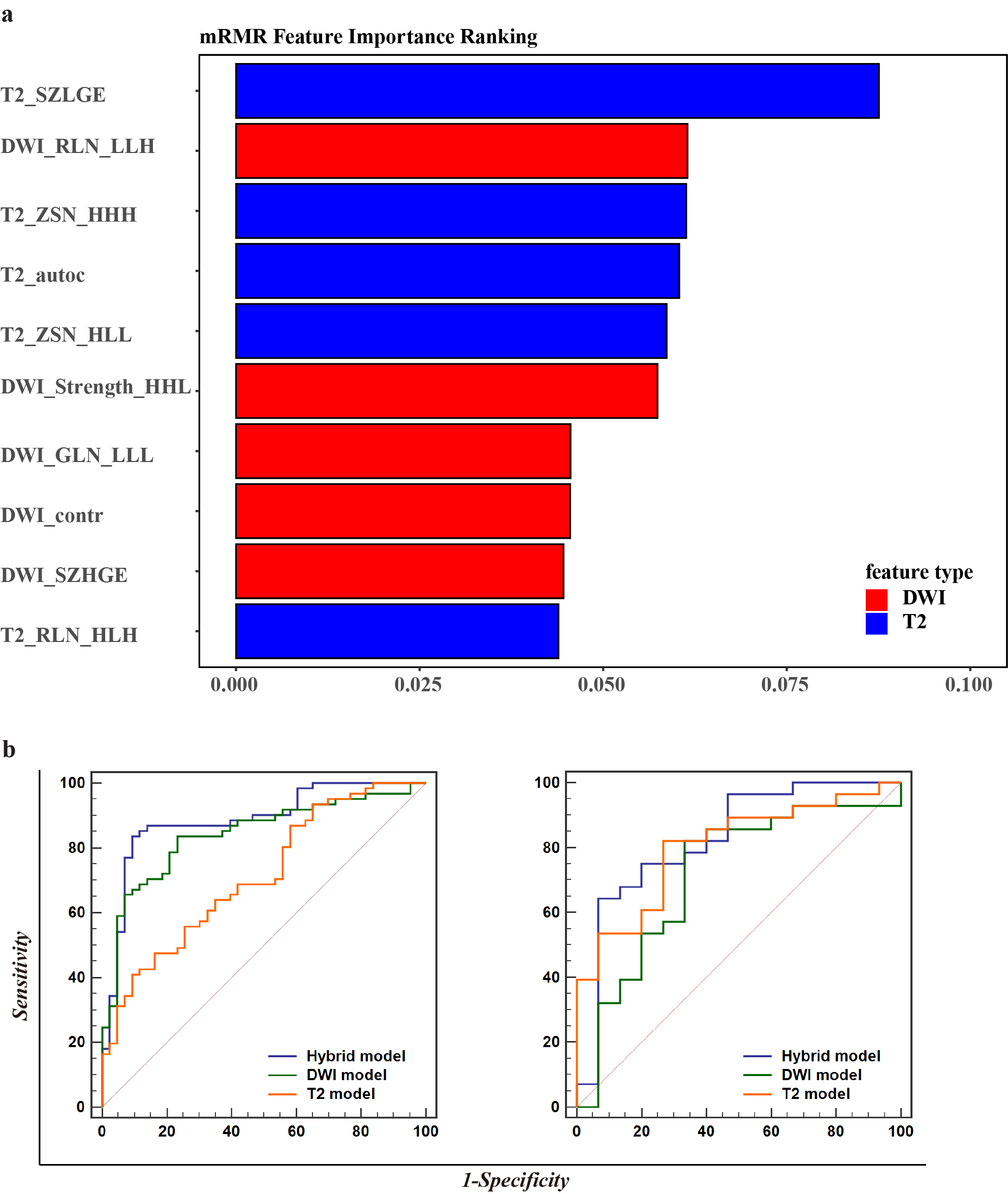

Supplement: Supplementary file 3 — Additional file 3:Supplementary Figure 2. Radiomics feature selection strategy and comparison of radiomics models. [file 43556_2023_133_MOESM3_ESM.tif]
